# Cost-Effectiveness Analysis of Acupuncture, Counselling and Usual Care in Treating Patients with Depression: The Results of the ACUDep Trial

**DOI:** 10.1371/journal.pone.0113726

**Published:** 2014-11-26

**Authors:** Eldon Spackman, Stewart Richmond, Mark Sculpher, Martin Bland, Stephen Brealey, Rhian Gabe, Ann Hopton, Ada Keding, Harriet Lansdown, Sara Perren, David Torgerson, Ian Watt, Hugh MacPherson

**Affiliations:** 1 Centre for Health Economics, University of York, York, United Kingdom; 2 Department of Health Sciences, University of York, York, United Kingdom; 3 Hull York Medical School, University of York, York, United Kingdom; Johns Hopkins Bloomberg School of Public Health, United States of America

## Abstract

**Background:**

New evidence on the clinical effectiveness of acupuncture plus usual care (acupuncture) and counselling plus usual care (counselling) for patients with depression suggests the need to investigate the health-related quality of life and costs of these treatments to understand whether they should be considered a good use of limited health resources.

**Methods and Findings:**

The cost-effectiveness analyses are based on the Acupuncture, Counselling or Usual care for Depression (ACUDep) trial results. Statistical analyses demonstrate a difference in mean quality adjusted life years (QALYs) and suggest differences in mean costs which are mainly due to the price of the interventions. Probabilistic sensitivity analysis is used to express decision uncertainty. Acupuncture and counselling are found to have higher mean QALYs and costs than usual care. In the base case analysis acupuncture has an incremental cost-effectiveness ratio (ICER) of £4,560 per additional QALY and is cost-effective with a probability of 0.62 at a cost-effectiveness threshold of £20,000 per QALY. Counselling compared with acupuncture is more effective and more costly with an ICER of £71,757 and a probability of being cost-effective of 0.36. A scenario analysis of counselling versus usual care, excluding acupuncture as a comparator, results in an ICER of £7,935 and a probability of 0.91.

**Conclusions:**

Acupuncture is cost-effective compared with counselling or usual care alone, although the ranking of counselling and acupuncture depends on the relative cost of delivering these interventions. For patients in whom acupuncture is unavailable or perhaps inappropriate, counselling has an ICER less than most cost-effectiveness thresholds. However, further research is needed to determine the most cost-effective treatment pathways for depressed patients when the full range of available interventions is considered.

## Introduction

Depression has the fourth highest burden of disease, and is expected to have the highest in high-income countries by 2030 [1]. In England an estimated 2.6 million cases of depression are reported with an economic burden estimated to exceed £9 billion per annum, with approximately £370 million covering direct costs of treatment [2]. However, up to 33% of patients do not show an adequate response to pharmacological antidepressant treatment,[3] and 30% do not adhere to their medication regime [4].

A number of non-pharmacological high-intensity psychological interventions are available for the treatment of moderate to severe depression, or mild depression with inadequate response. The United Kingdom’s (UK) National Institute for Health and Care Excellence (NICE) clinical guidelines recommend cognitive behavioural therapies (CBT), interpersonal therapy (IPT), behavioural activation (ACT) and behavioural couples therapy [5]. Overall, NICE found the evidence for counselling to be limited, however, counselling is recommended by NICE for patients who have declined antidepressants, CBT, IPT, ACT and behavioural couples therapy. For a person whose depression has not responded to either pharmacological or psychological interventions, the clinical guidelines recommend combining antidepressant medication with CBT, for which evidence suggests that combined CBT/medication is more effective and cost-effective than either treatment alone [5]–[7]. However, a more recent meta-analysis found that all seven psychotherapeutic interventions examined were more effective than usual care. The only significant difference between interventions was that IPT achieved an improved effect compared with supportive counselling. [8] Counselling is widely used for patients with depression, with 9000 primary care practices in England offering referrals [9] despite its limited recommendation [5].

Acupuncture is provided as a treatment most commonly for chronic pain[10], for which there is evidence of a beneficial effect [11]. Until recently the evidence on the effectiveness of acupuncture for depression has been found to be inconclusive [12], although new evidence of clinical benefits have been recently reported in the ACUDep trial, in which acupuncture and counselling were compared with usual care for patients with on-going depression. [13] Acupuncture is rarely provided within the UK’s mental health service or primary care, but private provision of acupuncture for depression is not uncommon [10].

This study explored the cost-effectiveness of acupuncture plus usual care (acupuncture), counselling plus usual care (counselling) and usual care alone using the health economic findings of the trial: Acupuncture, Counselling or Usual care for Depression (ACUDep) (ISRCTN63787732). The clinical findings have been reported previously [13]. In the clinical report of the ACUDep trial, patients in both the acupuncture and counselling arms showed improved depression scores on the primary outcome, the Patient Health Questionnaire (PHQ-9) scale[14], compared with usual care alone at 3 and 6 months as well as in an area-under-curve analysis over 12 months. There were no statistically significant differences between the counselling and acupuncture arms.

Given the positive clinical results in the ACUDep trial, the primary aim of this study was to assess the health-related quality of life and resource use reported in the trial to determine the cost-effectiveness of short courses of acupuncture or counselling compared with usual care alone for patients with moderate to severe depression.

## Methods

### Trial

ACUDep was an open parallel-arm randomised controlled trial with patients randomised to one of three arms using the allocation ratio of 2∶2∶1, respectively: 12 weekly sessions of acupuncture; 12 weekly sessions of counselling; and usual care alone. The pragmatic design meant patients were not restricted from receiving interventions associated with the trial groups in which they were not randomised or other types of psychological interventions. Patients were eligible if they were 18 or over, had consulted with depression in primary care within the past 5 years and who were continuing to experience moderate to severe depression based on a score of at least 20 on the Beck Depression Inventory (BDI-II) [15]. Further eligibility criteria have been described elsewhere [13], [16].

### Health Outcomes

The measure of health benefit used in the economic analysis was the quality adjusted life year (QALY), which takes into account the treatment differences in health-related quality of life (HRQoL) and mortality. HRQoL was measured using the EuroQol (EQ-5D) instrument, at baseline, and at months 3, 6, 9 and 12 in ACUDep. The EQ-5D measures health-related quality of life on five dimensions (mobility, self-care, usual activities, pain/discomfort and anxiety and depression). Each dimension is subdivided into three levels which corresponded to whether a respondent has no problems, moderate problems or extreme problems (Table S1: EQ-5D Level Descriptions). The value of each of the 243 unique health states is preference weighted using valuations from a UK population [17]. The EQ-5D is preferred by NICE for assessing cost-effectiveness [18].

### Costs

A National Health Service (NHS) cost perspective was used, although out-of-pocket expenses were also reported.

Resource use data were collected at 3, 6, 9 and 12 months using patient questionnaires in the trial. Patients were asked how many times they visited different health care providers over the last 3 months for “any reason” (i.e. the total number of visits to a provider) and “about your depression only” (i.e. depression related visits). Total annual resource use was calculated as the sum of the resource use collected at each 3-month period. The total annual cost was calculated by multiplying the total annual resource use by publicly available 2012 national unit costs (Table S2: Unit Costs). As the cost of acupuncture is not currently financed by the NHS, we used the costs of acupuncture as estimated previously and the average of the ranges reported by NHS Choices, £47.50, for an initial session and £37.50 for subsequent sessions [19], [20]. The costs of counselling are those currently used in the NHS, £65 per hour of client contact [21]. Total annual costs were missing for many patients, due to missing resource use data at one or more follow-up periods.

### Statistical Analysis

Multiple imputation methods were used to manage the uncertainty caused by the missing data. Chained imputation using predictive mean matching was undertaken using resource use data, PHQ-9 and BDI scores, QALYs, and patient characteristics such as age, sex and education.

EQ-5D data were analysed using ordered logit models on each of the five dimensions of the instrument. Analysis at 3 months controlled for the baseline response and analysis over 12 months used random effects models and controlled for the baseline response and the timing of each response (i.e. the day from randomization).

HRQoL weights were calculated using an independent predefined algorithm obtained by the elicitation of societal preferences for EQ-5D health states in a random population sample through a time trade-off technique. The UK valuation of the EQ-5D results in a scale from −0.594 to 1, where negative numbers represent states worse than death, 0 represents death and 1 represents perfect health [17]. QALYs were calculated by applying an individual’s HRQoL weights and the time between EQ-5D measures using the area under the curve approach [22], [23]. For all cost-effectiveness analyses seemingly unrelated regressions were used to account for the correlation between costs and QALYs [24]. QALYs were regressed on the base line HRQoL and treatment arm, and costs were regressed on the treatment arm only.

Incremental cost-effectiveness ratios (ICERs) were estimated using fully incremental analysis. We did not consider pairwise comparisons appropriate given the economic requirement to include all relevant comparators and because pairwise comparisons may lead to misleading conclusions, for example, if ICERs are calculated between treatments when one of the treatments is dominated by a third treatment not included in the calculation.

### Sensitivity Analysis

Base case results were calculated using total costs and taking into account the uncertainty from the multiple imputation and the seemingly unrelated regression. Probabilistic sensitivity analysis was used to reflect uncertainty in mean total costs and QALYs and we estimated the probability of cost-effectiveness conditional on alternative cost effectiveness thresholds. Further exploratory scenario analyses were undertaken to understand the influence on cost-effectiveness of (i) the differential cost of the acupuncture and counselling interventions, (ii) depression related resource use (i.e. those visits determined by the patient to be related only to depression), (iii) complete case and (iv) a population for which acupuncture is not appropriate or unavailable (e.g. those with needle phobia).

### Cost-effectiveness thresholds

All analyses considered the published NICE cost-effectiveness thresholds of £20,000 and £30,000 per QALY gained [18]. Additionally, we considered a recent empirically estimated NICE threshold of £13,000 per QALY [25]. These thresholds are meant to represent the opportunity costs of the NHS. Thus, ICERs below the threshold suggest that the intervention is a good use of NHS resources, while ICERs above the threshold provide less health than they displace.

## Results

### Health Outcomes

At 3 months patients treated with acupuncture or counselling were less likely than patients treated with usual care to report that they were moderately or extremely anxious or depressed rather than not anxious or depressed (Table 1). The 3-month improvement in anxiety and depression was sustained over the trial period to 12 months (Figure S1: Responses to the anxiety and depression dimension of the EQ-5D over 12 months and by treatment). At 3 months the direction of effect for the other EQ-5D dimensions is mixed; however, over the 12 months, the odds of being in the worse health states for all dimensions was lower for the acupuncture and counselling arms compared with usual care (Table 1).

**Table 1:** The proportional odds of being at level 2 or 3 compared with level 1* compared with usual care.

	At 3 Months		Over 12 Months	
EQ-5D Dimension	Acupuncture OR (95%CI)	Counselling OR (95%CI)	Acupuncture OR (95%CI)	Counselling OR (95%CI)
Anxiety and Depression	0.63 (0.40 to 0.98)	0.66 (0.42 to 1.02)	0.40 (0.23 to 0.70)	0.40 (0.23 to 0.70)
Pain	0.77 (0.48 to 1.23)	0.96 (0.60 to 1.53)	0.87 (0.49 to 1.54)	0.88 (0.5 to 1.55)
Usual Activities	1.14 (0.48 to 2.71)	1.05 (0.44 to 2.54)	0.57 (0.34 to 0.95)	0.72 (0.43 to 1.21)
Self-care	0.81 (0.52 to 1.27)	0.85 (0.54 to 1.33)	0.40 (0.15 to 1.09)	0.58 (0.22 to 1.53)
Mobility	1.29 (0.64 to 2.61)	1.19 (0.59 to 2.41)	0.89 (0.41 to 1.94)	0.74 (0.35 to 1.59)

*Levels 1–3 represent low, moderate and high disability respectively. For more detailed information on EQ-5D levels see Table S1.

The odds ratios below 1 indicate that the treatment was correlated with fewer patients reporting being in the more severe health states than patients in the usual care arm, i.e. the OR 0.63 in column 2 suggests that patients in the acupuncture arm were less likely to report being moderately or extremely anxious or depressed than patients in the usual care arm at 3 months. The odds ratios above 1 suggest that the treatment is correlated with more patients reporting being in the more severe health states than patients in the usual care arm.

**Figure 1:**
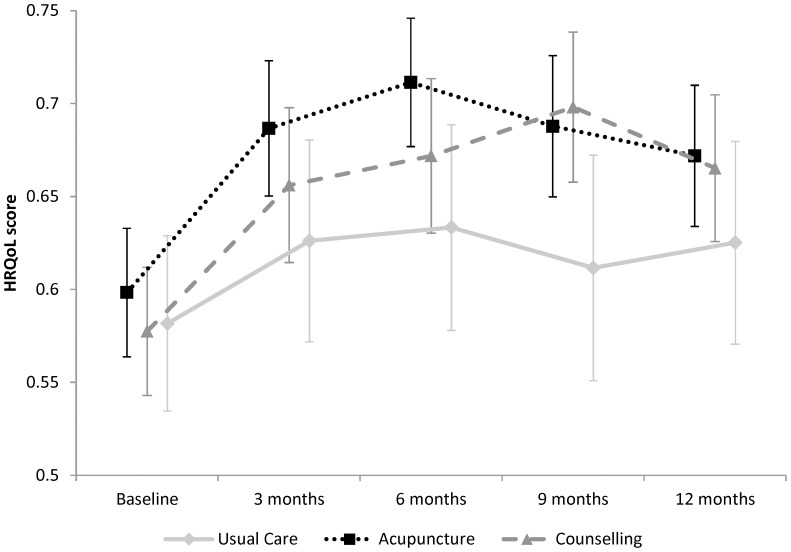
Health-related quality-of-life scores over time and by treatment.

Combining the EQ-5D dimension results with the UK population health state preferences resulted in the HRQoL scores over time and by treatment presented in Figure 1. For all treatment arms the HRQoL increased between baseline and 3 months with acupuncture and counselling arms being higher than usual care and remaining higher at 12 months.

QALYs for usual care, acupuncture and counselling were estimated to be 0.604, 0.663 and 0.666, respectively, using imputed data and seemingly unrelated regression controlling for the baseline HRQoL.

### Costs

Mean NHS resource use was highest for patients in the usual care group (Table 2). The sample of patients reporting resource use was small, but complete case results were similar to the imputed results. Total costs and depression related costs were reported in Table 3. As expected depression related costs were lower than the total costs. The higher resource use in the usual care group was offset by the additional costs of acupuncture and counselling sessions. Costs were lowest for patients in the usual care group and highest for patients in the counselling group. Differences between treatment arms were similar whether total or depression costs were used.

**Table 2:** Complete case and imputed mean number of service use contacts over 12 months.

Resource	Usual Care			Acupuncture			Counselling		
	N	Completecase mean(95%CI)	Imputedmean(95%CI)	n	Completecase mean(95%CI)	Imputedmean(95%CI)	n	Completecase mean(95%CI)	Imputedmean(95%CI)
GP	69	6.48	6.56	145	5.57	5.66	127	4.94	5.06
		(5.16 to 7.80)	(5.37 to 7.75)		(4.78 to 6.37)	(4.88 to 6.43)		(4.19 to 5.7)	(4.38 to 5.73)
Practice Nurse	60	1.40	1.53	140	1.16	1.25	127	1.25	1.36
		(0.86 to 1.94)	(1.05 to 2.02)		(0.84 to 1.48)	(0.95 to 1.54)		(0.95 to 1.55)	(1.05 to 1.66)
Other healthprofessional	54	1.39	1.76	133	1.24	1.37	116	1.4	1.54
		(0.74 to 2.04)	(0.93 to 2.6)		(0.7 to 1.79)	(0.85 to 1.89)		(0.78 to 2.01)	(1 to 2.07)
NHS hospitaloutpatient clinic	82	1.55	2.01	175	1.40	1.52	151	1.69	1.87
		(0.99 to 2.1)	(1.19 to 2.83)		(0.95 to 1.85)	(1.05 to 1.98)		(1.17 to 2.21)	(1.35 to 2.39)
Hospital ward	79	0.29	0.32	166	0.15	0.21	138	0.27	0.35
		(−0.01 to 0.60)	(0.04 to 0.6)		(0.02 to 0.28)	(0.05 to 0.38)		(0.06 to 0.47)	(0.12 to 0.59)
Hospital ICU	76	-	0	160	0.04	0.03	136	–	0
			(0 to 0)		(−0.04 to 0.11)	(−0.03 to 0.1)			(0 to 0)
Hospital mentalhealth unit	76	0.53	0.48	160	0.04	0.12	134	–	0.42
		(−0.48 to 1.53)	(−0.34 to 1.3)		(−0.04 to 0.13)	(−0.18 to 0.43)			(−0.13 to 0.97)
Other hospital unit	75	0.01	0.02	152	0.05	0.04	132	0.05	0.04
		(−0.01 to 0.04)	(−0.02 to 0.07)		(−0.04 to 0.14)	(−0.02 to 0.09)		(0 to 0.11)	(0 to 0.08)
Accident andemergency	84	0.37	0.40	191	0.21	0.25	157	0.35	0.35
		(0.17 to 0.57)	(0.15 to 0.64)		(0.13 to 0.29)	(0.13 to 0.37)		(0.11 to 0.59)	(0.16 to 0.53)
Community mentalhealth nurse	74	0.43	0.42	164	0.15	0.2	145	0.1	0.18
		(−0.04 to 0.91)	(0.04 to 0.79)		(0.02 to 0.29)	(0.04 to 0.37)		(−0.02 to 0.23)	(0.01 to 0.35)
Psychologist orpshychiatrist	72	0.75	0.73	164	0.21	0.31	135	0.35	0.55
		(0.04 to 1.46)	(0.16 to 1.3)		(0.08 to 0.34)	(0.09 to 0.54)		(0.09 to 0.61)	(0.22 to 0.88)
NHS counsellor notinvolved in the study	68	0.34	0.38	161	0.29	0.28	142	0.17	0.23
		(0.07 to 0.60)	(0.07 to 0.68)		(0.08 to 0.49)	(0.11 to 0.45)		(0.02 to 0.32)	(0.06 to 0.4)

**Table 3:** Complete case and imputed costs over 12 months.

Resource	Usual Care			Acupuncture			Counselling		
	N	Completecase mean(95%CI)	Imputedmean(95%CI)	n	Completecase mean(95%CI)	Imputedmean(95%CI)	n	Completecase mean(95%CI)	Imputedmean(95%CI)
Total costs (£)	22	621	958	69	1,110	1,227	59	1,355	1,450
		(365 to 877)	(739 to 1180)		(930 to 1291)	(1103 to 1350)		(1082 to 1627)	(1305 to 1592)
Depression relatedcosts (£)	18	226	496	54	769	913	48	962	1,006
		(92 to 360)	(288 to 704)		(644 to 894)	(764 to 1061)		(759 to 1166)	(761 to 1251)

Patients reported the amount spent on out-of-pocket acupuncture, counselling or therapy. Table 4 displays the means and standard deviations (SD) of out-of-pocket expenditures and days off work. Patients in the acupuncture arm reported spending the most on acupuncture while patients in the counselling arm reported spending the most on counselling and psychotherapy. The reported means for the number of days off work were similar across arms.

**Table 4:** Out-of-pocket costs and days of work for complete cases.

	Out-of-pocketacupuncture costs (SD)	Out-of-pocketcounselling costs (SD)	Out-of pocketpsychotherapy costs (SD)	Days off work(SD)
Usual Care	£6 (57)	£5 (32)	£3 (23)	231 (113)
	n = 98	n = 98	n = 82	n = 151
Acupuncture	£32 (93)	£6 (42)	£2 (33)	238 (115)
	n = 194	n = 188	n = 182	n = 302
Counselling	£7 (41)	£42 (173)	£15 (87)	240 (112)
	n = 169	n = 170	n = 157	n = 302

### Cost-effectiveness

When comparing acupuncture, counselling and usual care, acupuncture was found to be the cost-effective alternative with an incremental cost-effectiveness ratio of £4,560 per additional QALY compared with usual care alone with probabilities of being cost-effective of 0.68, 0.62 and 0.56 at thresholds of £13,000, £20,000 and £30,000 per QALY, respectively (Table 5). Counselling results in higher costs and benefits than acupuncture with an ICER of £71,757 per additional QALY compared with acupuncture.

**Table 5:** Incremental cost-effectiveness of Usual Care, Acupuncture and Counselling.

	QALY	Totalcosts (£)	ICER(£ per QALY)	Probability of Cost-Effectiveness		
				Threshold = £13,000 per QALY	Threshold = £20,000 per QALY	Threshold = £30,000 per QALY
Usual Care	0.604	958	–	0.07	0.03	0.02
Acupuncture	0.663	1,227	4,560	0.68	0.62	0.56
Counselling	0.666	1,450	71,757	0.26	0.36	0.42

A scenario analysis assuming each session of acupuncture is the same price as counselling (£65) resulted in counselling having higher QALYs and lower costs than acupuncture i.e. acupuncture was dominated (Table 6). Cost-effectiveness results were similar between the base case result using total costs and the scenario analysis using depression related costs only. Restricting the analysis to the complete case data resulted in an ICER for acupuncture of £10,979 per QALY and counselling having higher costs and lower QALYs than acupuncture. For patients in whom acupuncture is inappropriate or unavailable the incremental cost-effectiveness of counselling versus usual care was £7,935 per additional QALY.

**Table 6:** Incremental cost-effectiveness scenario analyses.

	QALY*	Totalcosts (£)	ICER(£ per QALY)	Probability of Cost-Effectiveness		
				Threshold = £13,000per QALY	Threshold = £20,000per QALY	Threshold = £30,000per QALY
i) Assuming acupuncture has the same cost as counselling (£65)						
Usual Care	0.558	524	–	0.15	0.06	0.03
Counselling	0.620	1,050	8,497	0.50	0.55	0.56
Acupuncture	0.617	1,073	Dominated	0.35	0.39	0.42
ii) Using depression related costs						
Usual Care	0.601	513	–	0.08	0.03	0.02
Acupuncture	0.659	853	5,819	0.61	0.58	0.54
Counselling	0.663	1025	50,612	0.32	0.39	0.44
iii) Complete case analysis						
Usual Care	0.638	648	–	0.43	0.29	0.20
Acupuncture	0.682	1,121	10,979	0.57	0.70	0.79
Counselling	0.643	1,378	Dominated	0.01	0.01	0.01
iv) Population for which acupuncture is not appropriate						
Usual Care	0.604	958	–	0.21	0.09	0.05
Counselling	0.666	1,450	7,935	0.79	0.91	0.95

*Some of the differences with the base case results (Table 4) are because of the probabilistic nature of the calculations.

## Discussion

The clinical results of the ACUDep trial demonstrated that acupuncture and counselling significantly reduced depression measures at 3 and 6 months when compared with usual care, as well as in an area-under-curve analysis over the 12-month period [13]. No statistically significant differences in clinical outcome between acupuncture and counselling were detected. This economic analysis demonstrated that the HRQoL results are consistent with the previously reported clinical results. NHS resource use was highest in the usual care group, but costs were highest in the counselling group followed by the acupuncture group because of the cost of the interventions.

The trial was powered based on the primary outcome of PHQ-9 and not the outcomes used in this analysis, EQ-5D and resource use. It is not surprising that the differences were not statistically significant at the standard p-values. Furthermore, inferential statistics are not helpful in making decisions about allocation of resources [26]. This study used probabilistic sensitivity analysis to incorporate the consequences of the variation in the trial results rather than using an arbitrary cut-off.

The cost-effectiveness results, taking into account the uncertainty in the estimates, suggest that acupuncture is the cost-effective option. Currently acupuncture for depression is not provided by the NHS. It is possible that the regulation of acupuncture may increase the per session costs. A sensitivity analysis was undertaken assuming that each acupuncture session costs £65, the same as counselling. In this scenario counselling is preferred to acupuncture because not only are the expected benefits higher but the expected costs are lower. This demonstrates that the cost-effectiveness of acupuncture in this study is reliant on having a lower price than counselling.

An analysis was undertaken comparing counselling to usual care alone to estimate the cost-effectiveness in patients for whom acupuncture is inappropriate. The trial was undertaken in a patient population able to undertake acupuncture thus this analysis assumes that costs and outcomes for counselling and the usual care alone arms in the trial will be the same for patients for whom acupuncture is not appropriate.

A cost-effectiveness analysis should consider the life-time horizon. In this trial patients were treated for up to 12 weekly sessions, although one patient in the counselling arm received 15 sessions, and patients outcomes were followed up for 12 months. The cost-effectiveness analysis considered a 12-month timeframe. This assumes that there are no differences in treatment arms past 12 months. This is expected to be a conservative assumption as there are expected to be no further intervention costs, but trial results suggest continued treatment differences at 12 months, although these treatment differences seem to be converging (Figure 1). Extrapolating these differences beyond 12 months would result in a lower ICER of acupuncture and no change to the conclusion.

When considering the possibility of incorporating further evidence into the analyses, only one previous trial of 59 patients with depression has been undertaken in the UK [27]. Patients received 12 sessions of acupuncture or sham acupuncture and outcomes were measured on the Beck Depression Index (BDI). The results of this trial were not included in this analysis due to the difference in the outcome used, the difference in comparator, the absence of health economic related data and the small number of patients included. Regarding counselling we found no trial based in primary care that evaluated counselling for moderate to severe depression.

Not all possible treatments for moderate to severe depression have been included in this analysis. Recent cost-effectiveness analyses on online cognitive behavioural therapy (CBT) report its cost-effectiveness versus usual care [5]–[7]. These analyses were based on the BDI rather than EQ-5D or PHQ-9, making comparisons with the current analysis difficult. It is also expected that some patients in the ACUDep trial may have received CBT as a part of usual care making the control group in this analysis different from previous trials. This study demonstrates that acupuncture is cost-effective compared with usual care alone given current levels and mixes of usual care but does not consider the alternative of improving usual care. Further analyses are needed to determine the cost-effectiveness of acupuncture and counselling when compared with other physical and psychological interventions as well as changing levels of usual care to understand how to best allocate scarce health care resources.

## Conclusion

The results of this analysis suggest acupuncture is cost-effective compared with counselling or usual care alone. This result is strongly influenced by the cost of acupuncture which only remains cost-effective when the cost of providing the intervention is lower than that of counselling. For patients in whom acupuncture is unavailable and perhaps inappropriate, counselling has an ICER less than a range of estimates of NICE’s cost-effective threshold. However, further research is needed to determine the most cost-effective treatment pathways for depressed patients when the full range of available interventions is considered.

## Supporting Information

Figure S1 **Responses to the anxiety and depression dimension of the EQ-5D over 12 months and by treatment.**
(TIF)

Table S1 **EQ-5d Level Descriptions.**
(DOCX)

Table S2 **Unit Costs.**
(DOCX)
