# Supplementary figures and images for: Cost-Effectiveness Analysis of Acupuncture, Counselling and Usual Care in Treating Patients with Depression: The Results of the ACUDep Trial
Source: PLoS One. 2014 Nov 26;9(11):e113726. doi: 10.1371/journal.pone.0113726 (PMC4245224; doi:10.1371/journal.pone.0113726)

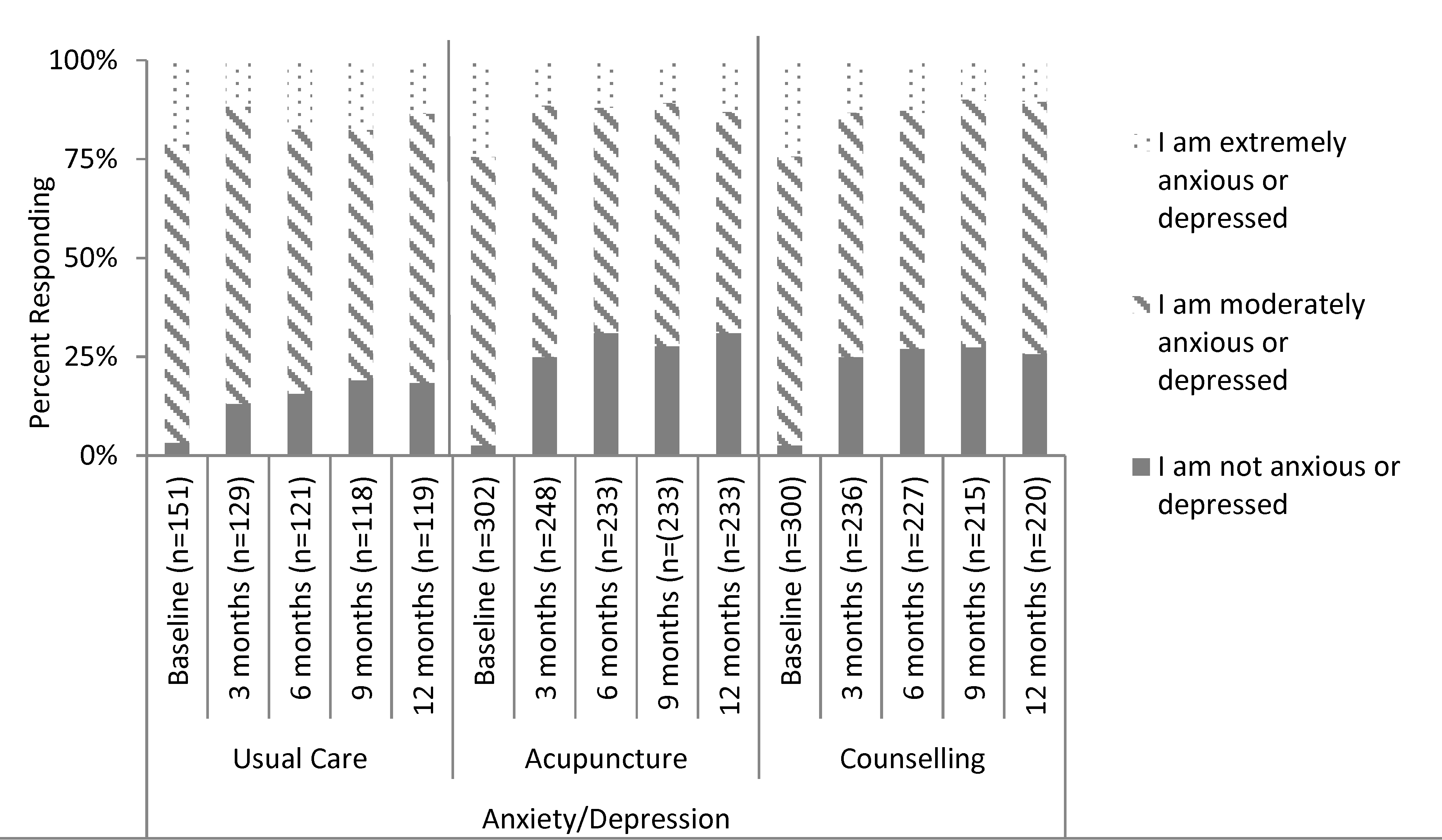

Supplement: Figure S1 — Responses to the anxiety and depression dimension of the EQ-5D over 12 months and by treatment. (TIF) [file pone.0113726.s001.tif]
