# Supplementary material for: Cost-Effectiveness Analysis of Acupuncture, Counselling and Usual Care in Treating Patients with Depression: The Results of the ACUDep Trial
Source: PLoS One. 2014 Nov 26;9(11):e113726. doi: 10.1371/journal.pone.0113726 (PMC4245224; doi:10.1371/journal.pone.0113726)
Supplement: Table S1 — EQ-5d Level Descriptions. (DOCX) [file pone.0113726.s002.docx]

Table S1: EQ-5D Level Descriptions

| EQ-5D Dimension | Level 1 | Level 2 | Level 3 |
| --- | --- | --- | --- |
| Anxiety and Depression | I am not anxious or depressed | I am moderately anxious or depressed | I am extremely anxious or depressed |
| Pain | I have no pain or discomfort | I have moderate pain or discomfort | I have extreme pain or discomfort |
| Usual Activities  (e.g. work, study, housework, family or leisure activities) | I have no problems with performing my usual activities | I have some problems with performing my usual activities | I am unable to perform my usual activities |
| Self-care | I have no problems with self-care | I have some problems washing or dressing myself | I am unable to wash or dress myself |
| Mobility | I have no problems in walking about | I have some problems in walking about | I am confined to bed |
