# Supplementary material for: Cost-Effectiveness Analysis of Acupuncture, Counselling and Usual Care in Treating Patients with Depression: The Results of the ACUDep Trial
Source: PLoS One. 2014 Nov 26;9(11):e113726. doi: 10.1371/journal.pone.0113726 (PMC4245224; doi:10.1371/journal.pone.0113726)
Supplement: Table S2 — Unit Costs. (DOCX) [file pone.0113726.s003.docx]

Table S2: Unit Costs

| Resource | Unit Cost (£) | Description | Source |
| --- | --- | --- | --- |
| Depression medication | 0.047 | Per dose of fluoxetine | BNF |
| Pain medication | 0.330 | Per dose of co-codamol | BNF |
| GP | 43 | Appointment of 11.7 minutes | PSSRU 2012 |
| Practice Nurse | 22 | Band 5, appointment of 15.5 minutes | PSSRU 2012 |
| Other health professional |  | Appointment of 11.7 minutes | PSSRU 2012 |
| NHS hospital outpatient clinic | 131 | Community setting mental health services | PSSRU 2012 |
| Hospital ward | 254 | Non-Elective Inpatient (Long Stay) Excess Bed Day HRG Data | NHS reference costs 2011-2012 |
| Hospital ICU | 654 | Mental health services inpatient attendances, intensive care - adult | PSSRU 2012 |
| Hospital mental health unit | 338 | Weighted average of all adult mental health inpatient bed days | PSSRU 2012 |
| Accident and emergency | 122 | Weighted average of “leading to admission” and “not leading to admission” | NHS reference costs 2011-2012 |
| Community mental health nurse | 85 | Band 5, per hour of client contact | PSSRU 2012 |
| Psychologist or psychiatrist | 136 | Band 8a, per hour of client contact | PSSRU 2012 |
| NHS counsellor | 65 | Band 7, per hour of client contact | PSSRU 2012 |
| Acupuncturist (initial session) | 47.5 | Average of initial sessions cost between £35 and £60 | BMC 2012 |
| Acupuncturist (further session) | 37.5 | Average of further sessions cost between £25 and £50 | BMC 2012 |
